# Hospital Meetings

**Published:** 1907-02-23

**Authors:** 


### HOSPITAL MEETINGS.

### CHARING CROSS HOSPITAL.

THE annual meeting was held on Wednesday, February 20, the Right Hon. the Earl of Kilmorey in the chair. The report shows an improvement in the financial position, largely due to the special Committee of Inquiry, presided over by Lord Sandhurst. It was decided to close two wards, containing thirty-seven beds. Three of the vacant wards have been let to the Royal National Orthopædic Hospital for one year and probably longer, all of which tends to diminish the deficit mentioned in the last report. A local maintenance fund has been inaugurated by the Earl of Kilmorey to make systematic efforts to obtain support from the neighbourhood in which the hospital works. The ordinary income last year amounted to £15,854, as compared with £11,911 in 1905, while grants and legacies and other funds brought it up to a total of £30,854. The ordinary expenditure was £18,770 last year, as compared with £20,111 in 1905, and while some part of the decrease is due to the closing of beds, part is due to economy, the cost per bed occupied having been reduced by £4.

The mortuary chapel and ante-chamber have been restored and furnished as a memorial to Miss Esther Hill, a brilliant pupil of the Royal College of Music.

### EAST LONDON HOSPITAL FOR CHILDREN.

THE half-yearly Court of the East London Hospital for Children was held on February 18. During the year 2,054 patients had been treated in the wards, an increase of 467 over last year, while 1,391 new cases were seen in the out-patients' department. The daily number of patients in the hospital averaged 113.34, and the average period of residence of each patient amounted to 21.38 days. By means of careful thought and economy on the part of the staff, the cost of maintenance of each patient per week had been reduced from £1 5s. to £1 0s. 6d.—a figure which might be regarded as almost a record in hospital administration. The increase in the number of in-patients was partly due to the extra number of beds obtained by the opening of the rest of the isolation block, but these beds were in use for only six months of the year. Their opening had, however, resulted in the wards enjoying a greater immunity from infectious diseases. The diphtheria ward and the two small wards for cases under observation had only been in use for six months, but the Board feels that their usefulness has already more than justified their cost.

There has been a decrease in annual subscriptions, which form the most reliable source of income. Donations showed a satisfactory rise, but notwithstanding this the financial position of the hospital, if it did not improve, will oblige the Board to consider some drastic measures of retrenchment. The Chairman felt that the existence and work of the hospital was insufficiently known, and suggested that a half-yearly Court, held at the Mansion House, might attract more attention to the needs of the institution. The Board congratulates the governors on the fact that, despite the great increase in the number of patients and the extra cost entailed by the maintenance of a new block for half the year, the total ordinary expenditure only exceeded that of last year by £50.

The Convalescent Home at Bognor has been much hampered in its work by continued outbreaks of infection and the necessary closing of the home for repainting, etc.

